# Visuomotor deficits during locomotion in previously concussed athletes 30 or more days following return to play

**DOI:** 10.14814/phy2.12252

**Published:** 2014-12-24

**Authors:** Carmen S. Baker, Michael E. Cinelli

**Affiliations:** 1 Department of Kinesiology and Physical Education, Wilfrid Laurier University, Waterloo, Ontario, Canada

**Keywords:** Collision avoidance, concussion, human locomotion, perception action integration

## Abstract

Current protocols for returning athletes to play (RTP) center around resolution of physical symptoms of concussion. However, recent research has identified that balance and cognitive deficits persist beyond physical symptom recovery. Protocols that involve testing dynamic balance and visuomotor integration have been recommended as potential tools for better understanding of length of impairment following concussion. A dynamic, visuomotor paradigm was undertaken in the current study to assess decision making in athletes who had sustained a concussion >30 days before study participation and had been cleared to RTP (*N* = 10). Two obstacles created a gap that varied between 0.6 and 1.8× participants' individual shoulder width in open space. Participants made decisions to navigate through or deviate around the gap created by the two obstacles. The results revealed that previously concussed athletes were highly variable in their decision making and demonstrated variable Medial‐Lateral (ML) center of mass (COM) control when approaching the obstacles, when compared with nonconcussed, age‐matched controls. As such, they showed poor visuomotor control and decision making, as well as poor dynamic stability compared to controls. Visuomotor deficits were persistent in the sample of previously concussed individuals, well beyond deficits identified by current RTP standards. This study suggests that dynamic, visuomotor integration tasks may be of benefit to increase rigor in RTP protocols and increase safety of athletes returning to sport.

## Abstract (precis)

Cognitive recovery from concussion does not coincide with physical symptom recovery. Deficits of concussion persist well beyond the believed 30 days of recovery. Visuomotor paradigms in combination with balance control can determine concussion recovery.

## Introduction

Current concussion management protocols for return to play (RTP) rely on self‐reporting of symptoms to determine progression of recovery. The RTP protocol recommends progression in a step‐wise fashion to more demanding physical and cognitive tasks. Progression is determined through the self‐reporting of physical symptoms, experienced by the concussed individual during the previous phase of recovery (McCrory et al. 2013).

However, two previous studies have found that athletes tend to falsify their results when asked to report their physical symptoms in order to return to sport competition (Ackery et al. 2009; Hollis et al. 2012). In these cases, the athletes return to activities prematurely, potentially exposing them to the harmful influences of multiple concussions or even second impact syndrome. Further, the Consensus Statement on Concussion in Sport only recommends multidisciplinary, conservative RTP for concussion cases with prolonged symptoms (>10 days; McCrory et al. 2013).

Physical symptoms of concussion resolve within days or weeks of injury (Iverson 2005), but the length of recovery from other concussion‐induced deficits (cognitive, balance, etc) is not well known, nor is concussion recovery known to correlate with physical symptom recovery. Broglio et al. (2007) investigated the relationship between resolution of symptoms and cognitive impairment in previously concussed varsity athletes. They compared cognitive scores (verbal memory, visual memory, visual‐motor speed, and reaction time) between baseline scores (before concussion) and after the athletes first became asymptomatic (average 8.14 days post concussion). The findings indicated that 38% of previously concussed athletes were impaired on at least one of the aforementioned cognitive measures (Broglio et al. 2007). In another study by Fazio et al. (2007) similar results were reported. These, and other studies, suggest that cognitive deficits of concussion persist beyond, and do not correlate with, physical symptom resolution (Parker et al. 2005; Catena et al. 2009; Sandel et al. 2012).

Current protocols that do assess deficits of concussion for RTP are limited to measures of reaction time, memory, and very simple decision‐making tasks. Balance coordination measures, if tested, are limited to static stability and simple upper limb tasks (i.e., reach and grasp; Locklin et al. 2010; Harmon et al. 2013; McCrory et al. 2013; Moy 2014). However, previously concussed athletes should be tested on sport‐specific tasks to ensure they have recovered the ability to perform dynamic stability, complex cognition, split‐second decision making, and visuomotor processing required of sport participation (Gérin‐Lajoie et al. 2007; Fajen et al. 2008; Higuchi et al. 2011). Measures of visuomotor processing have been suggested as a tool to identify persistent impairments following a concussion (Locklin et al. 2010). These vary in complexity and task requirements, and may identify concussion‐induced deficits better than current protocols.

Slobounov et al. (2006) administered a visuomotor task, in combination with balance control, to assess deficits of concussion. They induced a visual perturbation through a virtual reality version of the moving room paradigm (see Lee and Lishman 1975 for full details) and measured coherence between postural sway and visual scene oscillation at baseline, 10, and 30 days post concussion. The authors found reduced coherence, indicating poorer visuomotor integration, at 10 days and up to 30 days post concussion, suggesting that visuomotor deficits persist for much longer than physical symptoms. There was no significant difference between the baseline and 30 days measures, implying that visuomotor recovery had occurred at this time point (Slobounov et al. 2006).

Recovery from concussion may be better observed through more complex visuomotor tasks. In an experiment by Locklin et al. (2010) a Fitts' tapping task and the simple reaction time task from the CogSport© were administered to identify deficits in athletes <1 year post concussion (see Collie et al. 2003 for CogSport© reliability). The Fitts' task is a complex visuomotor integration task that employs varying target size as well as varying target distance (Fitts and Peterson 1964). These factors influence visuomotor processing in such a way that speed and/or accuracy of tapping decrease with decreasing target size and/or increasing distance. Ten varsity athletes who had sustained a concussion within the past year were compared to ten nonconcussed athlete controls, and ten nonconcussed, nonathlete controls (*N* = 10). No group differences were found on the reaction time task. Although nonsignificant as a group, previously concussed athletes tended to have poorer results on the Fitts' task than the control groups. Furthermore, half of the previously concussed athletes had slower movement times on the Fitts' task than either of the control groups. The authors suggest that increasing the complexity of the visuomotor task might identify significant impairments of concussion across the group (Locklin et al. 2010).

The purpose of this study was to identify visuomotor deficits following a concussion, beyond the typical recovery identified by RTP protocols. The current protocol utilized a visuomotor integration task in combination with dynamic balance control similar to that experienced during sport. It was hypothesized that, as the current task integrated dynamic balance control with complex visuomotor processes, deficits of concussion will be identified in athletes who are classified as being recovered.

## Methodology

### Participants

Ten previously concussed athletes (mean age =20.5 ± 1.65; four females and six males) volunteered for the study. Participants reported whether they were currently experiencing physical symptoms of concussion via the Sport Concussion Assessment Tool 2 Symptom Evaluation (2009). All participants had been cleared to RTP by a physician at the time of testing. All individuals were tested once, at approximately 30–110 days after sustaining their concussion (Table 1). All participants gave their written informed consent in accordance with the Wilfrid Laurier University Research Ethics Board. All testing procedures were approved by the Wilfrid Laurier University Research Ethics Board.

**Table 1.** Characteristics of participants recruited postconcussion. Current symptom severity was obtained through the sum of symptom severity of all reported symptoms prior to the testing session.

Gender	Age	Days post concussion	Current symptom severity	Duration of symptoms (days)	Time since symptoms abated (days)
F	20	32	0	1–3	29
M	19	34	19	1–3	31
F	21	38	0	1–3	35
M	20	48	0	4–7	41
F	18	77	2	>14	62
M	19	92	2	>14	77
M	21	95	0	4–7	88
M	21	98	0	4–7	91
F	23	101	6	4–7	94
M	19	109	12	>14	94

This study employed the same methodology and obtained data from nonconcussed Young Adults (*N* = 12, age: 23.8 ± 1.1 years; Hackney et al. 2013).

### Apparatus

**Figure 1.**
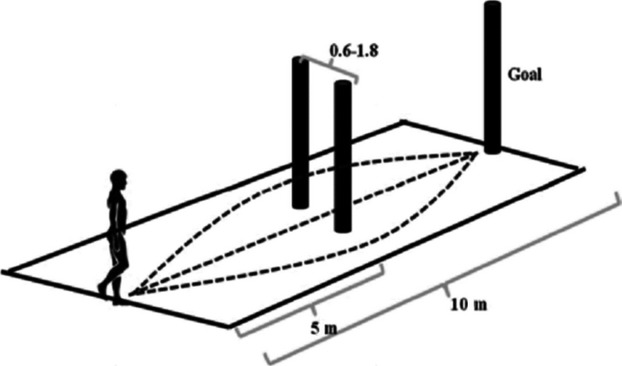
Experimental setup. Participants walked down a 10‐m‐long pathway. 5 m down the pathway two vertical obstacles created a horizontal gap along the midline of the room. The gap was scaled to 0.6–1.8× individual shoulder width. Participants had three choices for navigation around the obstacles: walk through or deviate around to the right or left of the gap.

The experiment was conducted within a 10 m by 6 m space. Participants walked along the long axis toward a vertically oriented goal (pole) at the end. Two identical pole obstacles (2.45 m tall × 0.17 m wide) were placed along the path on either side of the midline at 5 m from the start creating a gap (Fig. 1). The gap ranged between 0.6 and 1.8× (in increments of 0.2) each individual's shoulder width, on any given trial. Each participant's shoulder width was recorded by measuring the widest horizontal distance across the shoulders with a tape measure to the nearest 0.5 cm.

Kinematic data was collected using the OptoTrak camera system (Northern Digital Inc., Waterloo, ON, Canada) at a sampling frequency of 60 Hz. Participants were outfitted with seven Infrared Emitting Diodes, rear‐facing marker setup: three markers were placed on the head as a rigid body, one on both the left and right acromioclavicular joints, and one on both the 6th and 12th thoracic vertebrae.

### Procedure

During the experimental trials participants were instructed to “Walk at your normal pace along the path toward the goal and avoid colliding with the obstacles placed along the pathway”. No direct instructions were given for how to avoid the obstacles. Participants began each trial at one of four random starting locations (each location was separated by 20 cm in the anterior–posterior direction) to ensure that individuals were using visual information to guide their actions, rather than relying on a consistent number of steps to initiate a change in action. Before the start of each trial, participants were asked to avoid looking at the obstacles by looking down at their feet, while the experimenter changed the position of the obstacles, creating a new medial‐lateral gap between the obstacles. Each participant performed four baseline trials (no obstacles present) and 28 randomized experimental trials (seven gap widths × four trials). The baseline trials were used to determine each participant's straight walking path and strategy.

## Analyses and Results

**Figure 2.**
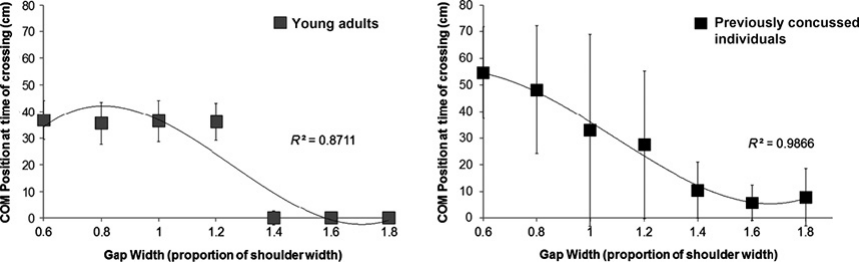
Medial‐Lateral center of mass (COM) position at the time of crossing (cm) is graphed for each gap width. Young adults (left) were found to walk through gaps that were 1.4× their shoulder width or greater, where previously concussed individuals (right) walked through gaps that were 1.0× their shoulder width or greater (*F*_1,9_ = 6.96, *P* < 0.05). Note the high variability within the previously concussed group particularly at gaps equal to 1.0× SW, or body size. This can be compared to the high consistency in COM position at each gap width for the young adult group.

Center of mass (COM) location in the anterior–posterior and medial‐lateral (ML) directions was calculated using a weighted average of the trunk marker similar to that used by Hackney et al. (2013). A between‐groups repeated measures analysis of variance (ANOVA) was conducted to determine the effects of gap size and group on decision making by comparing the ML position of the COM at the time of crossing the gap (distance from midline of path, cm). A main effect of gap size was found (*F*_3,2_ = 35.9, *P* < 0.05), where ML COM position for gaps 0.6 (43.7 ± 16.2), 0.8 (40.6 ± 18.1), and 1.0 (31.7 ±24.4) times participants' shoulder width were significantly greater than gaps 1.2 (21.6 ± 21.9), 1.4 (6.6 ± 10.7), 1.6 (2.9 ± 5.3), and 1.8 (3.9 ± 8.1) times participants' shoulder widths. This indicates that collectively, individuals tended to walk around gaps that were smaller than 1.2× their shoulder width, and walk through gaps equal to or >1.2× their shoulder width. A main effect of group was also observed (*F*_1,9_ = 6.96, *P* < 0.05). Pairwise comparisons revealed that young adults walked through gaps that were 1.4× their shoulder width or greater (*P* < 0.05) whereas previously concussed athletes tended to walk through gaps that were 1.0× their shoulder width or greater (*P* < 0.05; Fig. 2). Therefore, previously concussed athletes showed less cautious behavior than their nonconcussed counterparts. However, previously concussed athletes were highly variable within this measure (large standard deviation; Fig. 2), and this variability is the greatest at gaps equivalent to their shoulder width (32.9 ± 36.1). When coefficient of variation was analyzed through a repeated measures ANOVA, a main effect of gap width was found (*F*_3.12,9_ = 16.25, *P* < 0.05). Pairwise comparisons revealed previously concussed athletes were more variable at gaps of 0.8, 1.0, 1.2, and 1.4× shoulder width (SW) when compared with gaps of 1.8× SW (*P* < 0.05).

Further analyses were conducted to determine if dynamic balance control (i.e., ML COM variability during the first 3 m of approach) correlated with visuomotor performance (i.e., ML COM variability at the time of crossing the obstacles). A larger variability in ML COM position (sway) during the approach indicated poorer dynamic stability whereas a larger variability in ML COM position at the time of crossing indicated more variable visuomotor performance (decision making). When collapsed across participants, Pearson's correlations revealed no relationship between ML COM position variability at the time of crossing and ML COM variability during the approach phase for individual gap sizes. Therefore, as a group, the level of control in dynamic stability during the approach does not influence the consistency in action strategy at the time of crossing the gaps across all conditions. When individual data points were used, positive correlations were found between ML COM variability during the approach and ML COM variability at the time of crossing. This was true for gaps equal to 1.0 (*r*_9_ = 0.59, *P* < 0.05), 1.2 (*r*_9_ = 0.63, *P* < 0.05), and 1.4 (*r*_9_ = 0.63, *P* < 0.05) times participants' shoulder width. Therefore, dynamic instability during the approach appears to be related to one's decision‐making capabilities at the time of crossing, suggesting that balance and decision‐making impairments were present in this previously concussed population.

We also tested whether physical symptom recovery was related to dynamic balance control or decision‐making capabilities. Pearson's correlations revealed that neither absolute recovery time (in days), recovery time since symptoms had abated (days), number of current symptoms, nor duration that symptoms persisted (days) correlated with ML COM position variability during the approach (*r*_9_ = 0.10, *r*_9_ = 0.13, *r*_9_ = −0.18, *r*_9_ = −0.38) or variability at the time of crossing (*r*_9_ = −0.07, *r*_9_ = −0.11, *r*_9_ = 0.03, *r*_9_ = 0.23). Therefore, no significant relationship was found between recovery from physical symptoms of concussion and dynamic stability or visuomotor deficits following a concussion.

## Discussion

The purpose of this study was to identify visuomotor deficits of concussion beyond that of the current standard assessment protocols for RTP (McCrory et al. 2013). Current standards of concussion assessment often do not measure visuomotor control (Locklin et al. 2010), although this is a vital aspect of sport that has been found to be impaired in individuals 30 days post concussion especially when combined with balance control (Slobounov et al. 2006; Fait et al. 2013; Howell et al. 2013a,b). This study employed a visuomotor paradigm through a choice navigation protocol.

The participants in this study were athletes who had previously sustained a concussion and had recovered for a minimum of 30 days, and had all been cleared for RTP by a healthcare professional. Overall, the results indicated that these individuals acted less cautiously than nonconcussed age‐matched controls (Hackney et al. 2013). They passed through gaps that were 1.0× their shoulder width or larger, whereas control subjects tended to pass through gaps that were 1.4× their shoulder width or greater (Fig. 2). Two possibilities may exist to explain these differences: (1) participants who had previously sustained a concussion were injured as a result of acting less cautiously than age‐matched nonconcussed young adults; or (2) participants who had previously sustained a concussion act less cautiously as a result of their concussion. It is important to note that these findings are likely not a result of athletic training, as no significant differences were observed between action strategies of specifically trained athletes and nonathletes under the constraints of this paradigm (Hackney et al. 2015). Both of the above explanations suggest that previously concussed athletes are at an increased risk of reinjury. This is particularly dangerous as it increases the likelihood of another concussion or even second impact syndrome, a condition that leads to devastating cognitive deficits, and potentially, death (Dessy et al. 2014).

**Figure 3.**
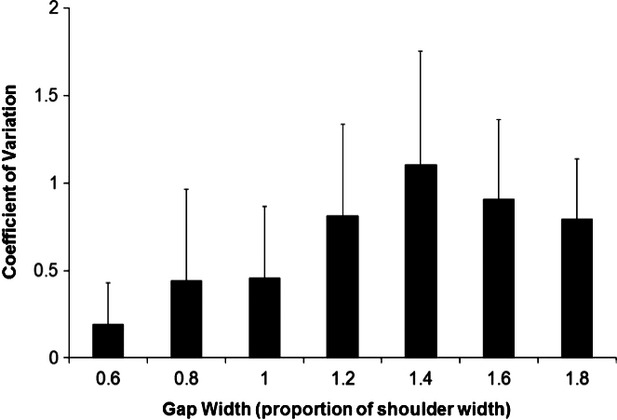
Coefficient of variation in medial‐lateral center of mass (COM) position at the time of crossing was determined to normalize standard deviation across all conditions (SD/AVG). Results are reported for the previously concussed group only. Results identify that previously concussed individuals demonstrate the highest variability in path choice at gaps equal to 1.4× SW. Such results suggest that previously concussed individuals struggle with decision making at the previously reported critical point of young adults (Hackney et al. 2013).

Results of this study also suggest that previously concussed athletes have less consistent action strategies that nonconcussed age‐matched controls (Hackney et al. 2013). Previously concussed athletes in this study were highly variable across gap widths 0.8–1.4× their shoulder widths (Fig. 3). It has been previously established that young adults are very accurate and consistent in their dynamic stability, visuomotor strategies (Lee and Lishman 1975; Patla 1997; Hackney and Cinelli 2013; Hackney et al. 2013), whereas individuals who have previously sustained a concussion demonstrate highly variable actions when required to perform visuomotor integration tasks (Slobounov et al. 2006, 2012; Fait et al. 2013). The present findings suggest that this variability continues beyond the identified recovery period in the aforementioned studies, as well as the current RTP protocols (McCrory et al. 2013).

Previous research conducted with this paradigm found that young, nonconcussed adults made consistent choices – as a group, and individually – to walk through gaps that were 1.4× their shoulder width or greater, and to walk around gaps that were 1.2× their shoulder width or smaller (Hackney and Cinelli 2013; Hackney et al. 2013). Although older adults have poor dynamic stability, they – as a group, and individually – consistently walk through gaps 1.6× their shoulder width or greater (Hackney and Cinelli 2013). Previously concussed athletes in this study acted inconsistently at the time of crossing the obstacles as a group (Fig. 3). The finding that previously concussed athletes were variable as a group is not surprising. Individuals in this study displayed different recovery times and recovery rates; group variability likely illuminates these individual differences. Yet, the previously concussed participants also demonstrated high variability individually. These results identify deficits in decision making and visuomotor integration, likely increasing the risk of reinjury upon returning to activity. Although previous research found that only 6.5% of NCAA football athletes had repeat concussions, the consequences of repeat injuries are catastrophic (Guskiewicz et al. 2003; McCrory et al. 2013; Dessy et al. 2014). Our results confirm the findings of Howell et al. (2013a,b) who identified the presence of postconcussive deficits up to 2 months after injury when assessed using a dual‐task, gait paradigm (Howell et al. 2013b). The findings of this study suggest a similar timeline of recovery, so our dynamic visuomotor integration task may be a useful indicator of concussion recovery, but this needs to be established in a larger cohort of athletes.

Our study identified a potential relationship between dynamic instability and poor decision making. ML COM variability during the approach to the obstacles positively correlated with the variability in ML COM position at the time of crossing. Greater variability in ML COM during straight walking is indicative of poorer dynamic stability. On the other hand, greater variability in ML COM position at the time of crossing might indicate either poorer decisions in action (i.e., pass through the gap on some trials and circumvent other similar gap sizes) or poorer control of body position relative to the obstacles at the time of crossing. Although it is possible that due to the small sample size, a few cases drove this relationship, such findings must be noted and further investigated to ensure the safety of athletes for RTP.

Interestingly, findings from this study suggest that the participants behaved most inconsistently with apertures that were the greatest threat to stability, between their own shoulder widths (1.0) and the critical point of their nonconcussed counterparts (1.4; Figs 2, 3). This suggests that previously concussed athletes have difficulty in making consistent action choices and controlling their body position even after concussion‐related symptoms have resolved. Therefore, it is likely that dynamic instability and decision‐making deficits of concussion are related, and persist well beyond 30 days post concussion. This finding was independent of duration since symptoms abated, as well as number of current symptoms, suggesting that recovery is specific to the individual. However, further research is required to confidently conclude the lack of this relationship.

To align with previous RTP assessments, correlations were run to determine if dynamic stability and visuomotor deficits were related to physical symptom recovery following a concussion. No significant correlations were found between dynamic stability and amount of recovery time since symptoms had abated, amount of absolute recovery time, number of current symptoms, or the duration symptoms persisted. As such, physical symptom recovery, the measure for RTP status, was not found to relate with visuomotor deficits we observed. These results are not surprising as numerous studies have failed to evidence a relationship between recovery from physical symptoms of concussion and recovery of balance or cognitive abilities (Parker et al. 2005; Broglio et al. 2007; Fazio et al. 2007; Catena et al. 2009; Sandel et al. 2012). Our results mirror the understanding that physical symptom recovery is not indicative of absolute recovery from concussion. Therefore, measuring ability through more complex, dynamic skills such as our dynamic visuomotor task are likely better predictors of concussion recovery.

This study did not attempt to replace the current standards of concussion assessment, which are currently the most accurate measures of evaluating individuals for RTP. However, the current standard assessments may not identify concussion‐related deficits with enough rigor to safely assess RTP (Fazio et al. 2007; Locklin et al. 2010; Hollis et al. 2012). This study identified poor decision making and reduced dynamic stability beyond 30 days post concussion, factors that may increase the risk of reinjury. These results suggest that dynamic visuomotor integration has not recovered in athletes who have been cleared for RTP. Therefore, the relationship between concussion recovery and dynamic visuomotor integration strategies, particularly related to sport‐related tasks, needs to be examined further.

This study implemented a visuomotor integration and decision‐making protocol that identified dynamic instability and deficits in consistent decision making related to concussion well beyond resolution of symptoms, well beyond the point that current standards had suggested that athletes were safe to RTP. The implementation of a visuomotor assessment with the inclusion of balance control to the current assessment battery may aid in returning concussed individuals to activity safely.

## Conflict of Interest

None declared.
